# New insight into the informal patients’ payments on the evidence of literature: a systematic review study

**DOI:** 10.1186/s12913-019-4647-3

**Published:** 2020-01-06

**Authors:** Arefeh Pourtaleb, Mehdi Jafari, Hesam Seyedin, Ali Akhavan Behbahani

**Affiliations:** 1 Health Management and Economics Research Center, Iran University of Medical Sciences, Tehran, Iran; 2 Health Managers Development Institute, Ministry of Health and Medical Education, Tehran, Iran; 3 Department of Health Services Management, School of Health Management and Information Sciences, Iran University of Medical Sciences, Tehran, Iran; 4 Department of Health in Disaster and Emergencies, School of Health Management and Information Sciences, Iran University of Medical Sciences, Tehran, Iran; 5 Instructor of Parliament Research Center, Tehran, IR Iran

**Keywords:** Informal payments, Health system, Content analysis, Systematic review

## Abstract

**Background:**

Nowadays, a growing literature reveals how patients use informal payments to seek either better treatment or additional services, but little systematic review has been accomplished for synthesizing the main factors. The purpose of this study was to analyze the content of literatures to demonstrate the factors for informal patient payments.

**Methods:**

In this systematic review study, PubMed, Web of Science, Wiley Online Library, Science Direct, Ovid, Scopus, and Iranian databases were investigated without time limitation for eligible English and Persian studies. Achieved data were analyzed using content analysis approach and MAXQDA _10_ software.

**Results:**

Themes related to informal payments in external context of health system were demographic features of health service consumers, patient’s personality features and social & cultural backgrounds of the community. Health system challenges’ themes were about stewardship weakness, and sustainable financing and social protection weakness. These were followed by human resources’ organizational behavior challenges, drugs, medical products, and services delivery provision process challenges and finally change management weakness for reducing and dealing with IPs.

**Conclusion:**

It appears that improving the quality of health care services and accurate monitoring of delivery processes, along with performing some strategies for regulating payroll and medical tariffs, strict rules and regulations and improving health staff motivation, would be effective ways against informal payments. Improving the health insurance contribution, promoting transparency & accountability in health system especially in financing, identify precise control mechanism, using empower patient/public related approach, modifying community perception, reinforcing social resistance to unofficial payments and rebuilt lost social capital in health care are some of the other recommendations in this field. To practice these strategies, a comprehensive and systemic vision and approach is needed, however, the key point is that before applying any strategy the impact of this strategy on access, efficiency, equity, and other health systems’ goals and policies should be investigated due to the consideration.

## Background

Good health system is an essential issue for sustained economic, social development and decreasing the rate of poverty, but people need to be protected from being forced into poverty as a result of the cost of health care [1]. Health financing is critical for reaching Universal Health Coverage (UHC), but nowadays the financing mechanism, and also finding some ways for providing resources, repaying costs, and improving insurance coverage of the community have become major challenges in many health systems [2].

Financing is one of the four main functions of a health system along with stewardship, production of resources and provision of services [2]. The common methods for financing the health care services are taxes, social insurance, private health insurance, and out-of-pocket payment [3]. At the timed that a government is unable to properly finance healthcare system, the burden of funding will directly affect the people, and they have to pay for health services by themselves [4]. Direct payments made by individuals to health care providers at the time of service use is known as out-of-pocket payments (OOPs), which is the weakest and most unfair payment mechanism in health care system [5].

As governments look for new ways to expand access to health services with higher quality, decision makers are confronting IPs problems in all over the world [6]. IPs have been defined as a kind of OOPs, to individuals or health care organization out of formal payment channels, which could be in form of cash, presents, gratitude aids, etc. These payments are widespread and frequent, as the main financial resources for health care services, especially in many transitional and developing countries [7, 8] that cause serious barriers in health system reform [9–12]. The IPs’ frequency range is enormous: from 3% in Peru to 96% in Pakistan. Regionally, South Asia stands out for its heavy reliance on IPs. East Asian experience is split between Thailand and Indonesia, with low levels, and the former Communist countries, with Cambodia at 55%, and also estimating 81% for Vietnam [7]. In six Central and Eastern European countries approximately 35–60% of the population experienced IPs for health services at least once in each country [13]. In 2017, another study indicated that the lowest and the highest scale of IPs were in Slovenia 2.7% and Azerbaijan 73.9%, respectively [14]. This phenomenon is also prevalent in Africa and South America [15].

Earlier studies proved that one of the drawbacks of Iran’s health system is unofficial payments [16–19]. Accordingly, Iran’s Ministry of Health and Medical Education has begun a series of reforms under the ‘Health Transformation Plan’ (HTP) since 2014, with a particular focus on financial protection. This plan consists of eight service packages, and one of its main goals is to reduce IPs or eliminate it from the health system that cause serious barriers in health system reform [20]. But it has been confirmed that, IPs are still prevalent for both outpatient and inpatient services and reform failure in eradicating IPs [21, 22]. In 2019, Meskarpour et al. demonstrated that about 27.7% of Iranian population had at least one IPs experience for health services and the prevalence rates of compulsory and voluntary IPs were 21.4 and 11.5%, respectively. In addition, IPs were reported to be 26.1% in inpatient and 12.5% in outpatient wards [21].

IPs for health services is considered as a main topic for researchers in all over the world [23–27], but little has been performed in terms of systematic reviews to synthesize the reasons for this complex phenomenon. The purpose of this study was to analyze the content of these literatures to demonstrate the factors for informal patient payments in health services. This can be happened to cover the costs of health care and to obtain advantages to other patients. Our focus in this study is only on informal patient payments and do not include kickbacks or other such payments paid by pharmaceutical or medical companies to medical professionals.

## Methods

### Databases and search strategies

In this systematic review, PubMed, Web of Science, Wiley Online Library, Science Direct, Ovid, Scopus, and Scholar along with some Iranian databases such as SID and Magiran were investigated. The key words were “informal payment”, “unofficial payment”, “under the table payment”, “under the counter payment”, “illegal payment”, “bribe”, “gift” and “gratitude” in title and abstract. No time limitation was considered for retrieving studies. Primarily, a study protocol, which consisted of formulating the study question, defining the inclusion and exclusion criteria and developing a database search strategy, was developed. The stages of the other study included retrieving the relevant studies, extracting the relevant data, appraising the retrieved studies, synthesizing the data and creating a report. The reference lists of the retrieved articles were scanned for any relevant papers that were not found in the first search. Furthermore, hand searches were performed for identifying additional studies and completing the search coverage.

### Inclusion and exclusion criteria

The inclusion criteria were: studies published in English or Persian languages; conducted by applying qualitative, quantitative, or mixed method designs; and dealing with either empirical or theoretical aspects of IPs. Also, those articles without full text and/or those that were duplicates, were excluded. Letters to the editors and conference presentations were excluded.

### Methods of screening and selection criteria

All the identified papers were imported to the Endnote software, and duplicate papers were deleted. All publications were qualitatively evaluated by two researchers who were experts in systematic reviews, and were also adequately familiar with the topic. Both researchers initially analyzed the titles for relevance. A consensus agreement was necessary for each publication to be documented on the review board. The relevance and role in developing the concept of the study were the main criteria for assessing the articles quality in the process of interpretive review of the articles [28].

Table 1 shows the quality appraisal checklist.

**Table 1:** Criteria for quality appraisal of the papers

Are the research goals and objectives clearly specified?	
Is the research design clearly specified and is it suitable for achieving research goals?	
Is the research process clearly explained?	
Are enough data displayed to support research interpretations and conclusions?	
Is the analysis method appropriate and adequately explained?	

*Source:* Dixon-Woods, et al. 2006 [29]

**Fig. 1:**
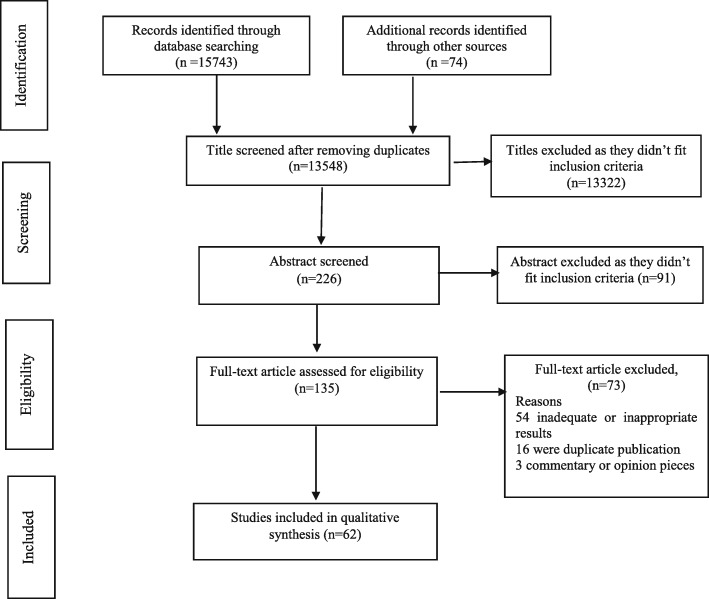
Literature review and retrieval flow diagram

Furthermore, the data associating with each of the selected papers was summarized in a form. Figure 1 explains the screening process of retrieved papers.

### Data analysis

In this qualitative systematic review, content analysis approach was used to summarize the data, this approach helps to explore and explain complex phenomena. Research questions exploring human experience are usually investigated through analyzing textual data collected in individual interview, focus groups, documents, or documented participant observation [31]. The initial step was read and re-read the texts to achieve a sense of the whole idea, and also to obtain a general understanding about what the authors talked about. After that, we started dividing the text into smaller parts, namely into the meaning units. These meaning units were labeled by formulating codes, and then these codes were classified into categories. According to the study aims, we went further and created themes for reporting the results. The coding was performed by 2 coders (AP and MJ), and Classes were identified based on the background knowledge of experts and researchers involved in the coding. This process was repeated until agreement about richness of classes was achieved. After that, one of the authors prepared a draft summary of all the studies’ findings, organized by theme. Later, two other review authors commented on this draft and finally they agreed on a final. The grouping of the codes was accomplished with respect to the World Health Report (2007) [32] and key issues from the literature review. We selected and modified this framework, as it was a popular and famous model about health system components. The approach of this framework is to define a discrete number of “building blocks” that make up the system based on their functions. The building blocks were service delivery; health workforce; information; medical products, vaccines and technologies; financing; and leadership and governance (stewardship) [32]. The coding was accomplished using MAXQDA Version 10 software. General characteristics of the included studies are shown in Table 2, and index of publications included in this review has been shown in Additional file 1. Also, the summary of literature review is described in Additional file 2.

**Table 2:** General description of included studies in terms of publication year, data collection year, and study wideness

Classification category	Sub-categories	N	Appendix-Index of publication
Publication year	1990–1995	0	–
	1996–2000	2	21, 58
	2001–2005	5	2,6, 12, 52, 57
	2006–2010	10	1, 20, 23, 30, 32, 38, 51, 54, 61, 62
	2011–2017	45	3,4,5,7,8, 9, 10, 11, 13,14, 15, 16, 17, 18,19, 22, 24, 25, 26, 27, 28, 29, 31, 33, 34, 35, 36, 37, 39, 40, 41, 42, 43, 44, 45, 46, 47, 48, 49, 50, 53, 55, 56, 59, 60
Data Collection year	1990–1995	1	58
	1996–2000	2	2, 57
	2001–2005	8	1,20, 23, 32, 39, 51, 52, 54
	2006–2010	20	3,4, 11, 14, 15, 16, 18, 19, 24, 26, 28, 29, 34, 35, 36, 43, 44, 46, 50, 62
	2011–2017	19	5, 7, 9, 10, 13, 22, 25, 27, 33, 37, 40, 45, 47, 48, 49, 55, 56, 59, 60
	Not clear	12	6, 8, 12, 17, 21, 30, 31, 38, 41,42, 53,61
Study Wideness	Single-country	45	1, 2,4, 5,6, 7,8,10, 11, 12, 14, 15, 17, 18, 19, 20, 22, 23, 24, 25, 26, 28, 29, 30, 32, 33, 35, 39, 40, 41, 42, 43,44, 46, 47, 49, 51, 52, 53, 54, 55, 56, 58, 59, 60
	Cross-countries	17	3,9, 13, 16, 21, 27, 31, 34, 36, 37, 38, 45, 48, 50, 57, 61, 62

## Results

The results’ tables consist of theme, category, and code headings in each column. All determinants were categorized in two large groups, which were called “external context of health system” and “internal context of health system”. Category and code in each part showed the key components and more details about the themes. External context of health system refers to what was not generally considered as a health system character. The result of external context of health system in this study is displayed in Table 3.

**Table 3:** Informal patient payment factors related to external context of health system

Theme	Category	Code
Demographic features of health service consumers	• Individual features • Household features	Age- sex- marital status- occupation- education- income level- residency- religious- race- health status- health insurance coverage- number of family members- socio economic situation of the family-
Patient’s personality features	• patient perception & attitude • Patient beliefs • Patient feelings • Patient willingness • patient relationships	Informal payment necessity- value of health issues in return for any payment- satisfaction- fear- value and respect for the doctor- more welfare- choosing the right doctor- Supporting the doctor- patient physician relationship- patient- staff relationships- relationship with other patient and their suggestion-
Social & cultural backgrounds of the community	• Low Community participation (Civil Society) • Low Public/ patient awareness • Value culture (normative) Corruption culture • Lost trust	Lack of media participation & social campaign- lack of public participation- lack of social planner’s participation- low knowledge & public awareness- low patient awareness about health care services - low awareness of patient rights- gratitude & tradition- compulsory social behavior- social attitudes and beliefs-culture of corruption in country - corruption in health system- governance corruption- lack of trust in political system and official strategies-lack of trust in government- lack of trust in insurance-

The external domain consists of three themes as followings: demographic feature of health services consumers, patient personality features and social and cultural background of the community. In “demographic feature of health services consumers”, we have two categories: individual features of patient and his/her household features, and both of them were explained by their own codes. Individual features of patient include demographic features of patient, while household features are associated with patient family aspects such as number of family members and socio-economic status of the family. The second theme in external context is “Patient’s personality features” and our literature review to syntheses items in this area forms five categories: patient perception & attitude, patient beliefs, patient feelings, and patient willingness and patient relationships. The other most important contributing theme for unofficial payments is social & cultural backgrounds in each community. Regarding, we found that the lost trust, corruption, value culture (normative), public/ patient awareness culture, and community participation are the leading factors of people decision for informally paying.

Table 4 shows the internal context of health system and related factors in this area. In our analysis, it was assumed that the internal context focuses on the weakness of health system functions and related issues in this field, which are commonly considered as the health system challenges and deficiencies.

**Table 4:** Informal patient payment factors related to internal context of health system

Theme	Category	Code
Stewardship weakness	lack of legal support towards IPs • Structural problems & inefficiencies of the health services providing network • Weak management • Lack of transparency & accountability • Lack of partnership and cooperation with other parts & stakeholders • Inefficiency in health information system	lack of regulation sanction and penalties for illegal behavior- Lack of regulation- lack of referral system-poor system design and structural weakness in providing services –duplication and fragmentation in health system- lack of control- limited management capacity- weak management in health system, hospital and medical center- related factors of resource allocation - less health insurance contribution- lack of private sector involvement- lack of support of health workers and professional association and their commitment or involvement-Lack of information- lack of data sources- lack of information on performance- lack of a comprehensive financial and tax reporting system-
Sustainable financing and social protection weakness	• Inefficient use of resources due to unfocused approach of funding activities and interventions for financing purposes • Lack of transparency in the health financing system • Insufficiency and inefficiency of the insurance system • Poor and vague definition of the basic benefit package	funding shortage in health system- inefficiency in financial management of health system- reliance on out-of-pocket payments- inefficiencies of payment mechanisms to providers- unrealistic, insufficient inequitable tariffs- Low income of doctors & medical personnel & deferred claims- inefficiency in financing health care system- insurance deduction & delayed reimbursement- Insurance coverage problems- Inadequate social protection for poor and other vulnerable groups
Human resources’ organizational behavior challenges	• Health workforces’ perception and motivation • Poor human resource management • Social position or authority • Moral/ ethical related issues	insufficient official income of health personnel and physicians-- lack of staff motivation- for higher standard of living in providers - lack of government attention to providers motivation- Imbalance in the distribution of medical staff - lack of control and monitoring on medical staff- misuse of monopoly power and market position- physician competency -IPs for well-known physicians- Lack of training in ethics- Low moral standards of the medical profession- low moral and ethical reasons-
Challenges of drugs, medical products and services delivery provision process	• The complexity & nature of the services • Organizational feature of the health services provider • inefficient patient complain process • Quality of health care services • Access to health care services • irrational prescription • Lack of medicine & other medical supplies	health workers providers specialty- length of stay- type of health services- kind of health services facilities- additional services- receiving special attention- better service quality- better care- skip waiting time- access to care -quick access to services- lack of access to health care services- scarcity of medicines and other supplies- irrational drug and treatment prescription- irrational diagnosis test prescription-
Change management weakness	• Lack of political will • Lack of follow up reform • Reform failure	lack of the reform monitoring- lack of range of policy tools in system for eliminating IPs-

With respect to the literature findings, the five main themes in the internal context of health system were governance weakness, sustainable financing and social protection weakness, human resources’ organizational behavior challenges, drugs, medical products and services delivery provision process challenges, and change management weakness. The stewardship weakness included lack of legal support towards IPs, structural problems & inefficiencies of health services providing network, weak management, less cooperation with other parts & stakeholders, lack of transparency & accountability, and inefficiency in health information system. Sustainable financing and social protection weakness theme concentrates on challenges in health system financing and the most important leading issues in this field that were mentioned earlier for IPs formation. It consisted of inefficient use of resources due to unfocused approach of funding activities and interventions for financing purposes, poor and vague definition of the basic benefit package, and lack of transparency in the health financing system. Due to the reason that insurance role and functions is basically related to health financing, consequently in this study, we put the health insurance position in health system financing part.

Among challenges of human resources’ organizational behavior, the main subjects were workforces’ perception and motivation, weak human resource management, social position or authority of medical personnel, moral/ ethical related issues of the health staff, and their medical education.

The categories in drug, medical products, and service delivery provision process challenges were about the complexity & nature of the health care services, organizational feature of the health services provider, insufficient patient complain process, and quality of health services and access to them. They were followed by irrational prescription and lack of medicine & other medical supplies that both can occur due to different reasons like induced demand, etc. Reform failure, lack of follow up reform, and lack of political were the most important topic dealt with IPs change management weakness.

## Discussion

The purpose of this systematic review study was to identify the contributing factors in IPs’ formation in health care services. Diverse ranges of objectives and scopes, research methods and results have been reported in this regard. A number of these studies have focused on the views and attitudes of patients and service providers toward IPs in health system [13, 17, 32–40], and a few of them have measured the impact of IPs on the health economy with respect to goals and indicators [41–45]. Several studies have focused on the definitions and methods of studying IPs and systematic reviews in this area [9, 46–48] . However, the main theme of the other studies was in regard with some factors affecting IPs [16, 49, 50]. Some of these studies were conducted at the level of a health care unit or one hospital in a limited geographic region [18, 51, 52], some at country level [53–56] , and a few cases were internationally performed across several countries [14, 57]. Due to specific and sensitive nature of IPs, it is difficult to study about it; however, all of these efforts have been carried out in an attempt to clarify the ambiguity surrounding the concept of unofficial payments in the health system. Briefly, the phenomenon of IPs is multi-causal and complex in nature and must be analyzed and addressed from different perspectives. Also, national and international studies have demonstrated that IPs exist in the community for various reasons including lack of government resources for healthcare financing, lack of trust and transparency in the health system, lack of adequate supervision in the system, low salaries and benefits for service providers, lack of proper accountability of service delivery, weak management, low-quality services, service receivers’ disappointment from insufficient health and welfare services, sociocultural factors, negligence in medical ethics, etc. [7, 8, 18, 46, 58].

Moreover, Findings confirmed that demographic features of health service consumers, patient personality features, and their social & cultural backgrounds are among the most important motivations for unofficial payment. In demographic features of health service consumers, patients’ ages appear to have a reverse association with IPs: in which that older people tend to have fewer IPs [51, 59–62] . Several studies have demonstrated that rural people are more willing to have IPs [7, 58, 59, 62, 63]. On the contrary, some studies have reported that rural people are less willing to make IPs because of their low-income levels [59, 61, 62] . However, it appears that disparate distribution of human resources is one of the noticeable challenges in this area, particularly in the medical profession. Studies on gender have indicated that women, in general, are more likely to pay informally [41, 54, 65, 66], which could be considered as a result of higher female referrals to doctors, and their need for more diagnostic and therapeutic services. Some studies indicated that household income does not affect unofficial payments [10, 67, 68] ; however, many studies indicated that prevalence and amount of payments are higher among individuals or families with higher incomes levels [10, 41, 59, 65, 67, 68]. The level of education also plays an unusual role in determining the amount of payments with contradictory findings regarding this issue. Some studies have indicated that the higher the level of education in patients, the lower is the incidence of IPs [19, 54, 60] , however in other studies; the results have demonstrated that higher levels of education increase unofficial payments. A comprehensive review has indicated that IPs occur in many countries, and also among different races and religions [69]. However, there would be many differences between socioeconomic and demographic factors that influence willing to pay, and amount and the frequency of payments. As the results indicated, patient perceptions and attitudes, patient beliefs, patient feelings, patient willingness, and patient relationships should be considered along with social & cultural backgrounds of the community. Literature reviews concerning IPs emphasized on these issues [41, 65–68]. It means that the person interprets his/her perceptions and assumptions in his/her own situation to have unofficial payments. In order to deal with challenges in this situation, we recommend patient centered approach, that emphasizing on patients’ preferences and concerns in health care delivery system. An alternate solution could be sufficient for availability of medical staff, improvement in personnel communication skills, responsiveness, and providing proper information to the patients [70, 71]. Consequently, it is important that the patient feels safe and guaranteed to receive a high-quality treatment especially in the event of a negative response to a subject request.

It has been stated that informal patient payment is an apparent sociocultural phenomenon that develops by passing the time [72, 73]. Lost trust, corruption, and value culture, public/ patient awareness and community participation were the common aspects of this complicate social phenomenon, which was proved in national and international studies [17, 18, 34, 37, 41] . It is necessary to increase the patient’s awareness about their rights, and also improve their participation in health policy making process and system reforms. Other strategies like better communication with the public particularly through media can help correcting the attitudes toward informal patient payments.

When it comes to IPs, although health system corruption manifests and remains as a main barrier to efficient and equitable health system, dealing with corruption in the public health system is pervasive and has increased over time. Findings proved that weak health system leadership and governance lead to arising different levels of corruption in health system, which represents another major contributing factor in justifying unofficial patients’ payments [18, 38, 74] . Some of these challenges in this field were lack of good governance [61, 69], lack of preventive laws and regulations and appropriate penalty system [13, 61, 65, 75], and lack of responsibility, transparency, and accountability in health system [65, 76, 77] and also structural problems [18, 27].

Finally, a contributory role of information is also proposed, as supported by other studies [78, 79], which are of great value for making transparency. Information is indispensable for effective management and development of health services, and therefore, it is considered as an important operational asset or resource. A Health Information System is mainly required to support management and operations at four levels as followings: transactional and functional, operational control, management planning and control and strategic planning. To provide the necessary information, a structured information system coupled with appropriate information technology is required. In addition, Adequate and relevant information is needed in terms of population characteristics, resources available and expended, output and outcome of health care activities. Additionally, information needs to be reliable, accurate, timely, easily accessible, and also indicating in a compact and meaningful form. By having a well-planned health information system, health authorities would be in a position to provide a quality, cost-effective and efficient health service for as many as people required, and also to provide optimal utilization of resources and to maintain and improve the community’s health status [80].

The result of this review demonstrated that factors related to health financing system were as followings: inefficient use of resources with an unfocused approach to funding activities, interventions for healthcare financing such as inefficiency in financial management of health system, reliance on out-of-pocket payments, inefficiencies of payment mechanisms to providers [13, 37, 55, 81], unrealistic tariffs [18, 63, 82], and inadequate social and financial protection against poor health system for people [68, 83, 84]. However, lack of transparency in the health financing system, and insufficiency and inefficiency of the insurance system were mentioned in other studies [47, 50, 56, 61, 85]. Insurance, as an absolutely crucial part of the health system, has an impressive effect on the incidence of illegal payments. A relationship between insurance coverage and reduction of IPs, or vice versa has been confirmed [61, 65, 81] . In addition, the delay in insurance reimbursement especially for physicians would increase IPs [41, 85, 86] . The low payments to health providers and low salaries of employees have led IPs to be considered as a collecting tool for additional funds (generally by medical organizations, and particularly by physicians) [85, 87]. However, most of the health sector reform measures attempt to address health-financing issues such as mobilization of funds, distribution of financial risks, allocation, services utilization, and provider payment incentives [88] . However, informal patient payments as a universal political issue could undermine government efforts in order to improve social justice, access to care, and anti-poverty policy [68, 89] . Consequently, it is important to investigate the health financing system and adopt some ways to finance the activities of the health sector. Furthermore, it is necessary to identify the patterns of financing in regard with the socioeconomic development, capacity and financial infrastructure, applicability, responsibility, and political accountability of health system, which following that, can be used for creating health policies oriented toward social justice.

Challenges of confronting human resources’ organizational behavior are other contributing factors to unofficial payments [51, 69, 90]. Health workforces’ perception and motivation, especially about salaries and reimbursement, is one of the main reasons for requesting or accepting IPs in cash or other ways [27, 37, 41, 53] , particularly for physicians, which is considered as a type of power and position abuse [6, 67, 85]. It has been stated that the government’s lack of attention to the economic status of healthcare providers [7, 91] along with their desire for a higher standard of life are among the reasons mentioned by healthcare providers to justify these types of income [12, 64] . Imbalance in the distribution of medical staff and lack of control and monitoring on medical staff are considered as the other reasons in this field. In addition, paying attention to proper management of human resources has been mentioned in studies. Regarding, an overview of human resource management in healthcare with special additional research on this topic is required for developing new human resource policies. Furthermore, more effective strategies for human resource management are greatly needed to attain better outcomes and access to health care systems.

Patients are routinely requested to pay for their medicine and other supplies required for their medical treatment, which was supported by number of studies [14, 27, 68] . Former studies indicated that the quality of provided services, type of services (hospitalization, outpatient cardiology services, maternity services, etc.), time, and access to health care services always were the main contributing factors to IPs [17, 18, 27, 51, 81, 92]. However, quality improvement and its assurance have been always emphasized in the health systems in all over the world, because of the vital nature of healthcare services and its close relationship with human wellbeing and life [93, 94]. Various options regarding this issue can be suggested, however it is better that healthcare officials rely less on personal knowledge, experience, and traditional management methods, and instead of that, the priority has to be given to necessity of applying new managerial techniques in order to provide superior health services to patients or consumers. In order to succeed in this field, performing appropriate, comprehensive, and effective strategies are needed, along with employee participation after considering their expectations and perceptions. These strategies can help increasing the productivity as well as facilitate the continuous improvement of healthcare processes and products, in a way that meet all customers` requirements and expectations.

### Limitations

Content analysis is one of the research methods in qualitative study, which is used for textual data analyzing. This approach focuses on the language characteristics as a communicating meaning in order to explore content and concept of the text. This study focus was just on English and Persian texts, as the researchers were fluent in both these languages; English as an international scientific language and Persian as the first language of the researchers’ team members, so other studies in other languages have not been considered in the analysis. The other limitation was that the discrimination between voluntary or compulsory payments and the different form of these payments has not been considered separately. As it was difficult to determine a clear boundary for payments to cover the costs of health care or to gain advantages to other patients, both purposes have been considered in this study.

## Conclusion

It can be concluded that, generally, IPs has significant negative impacts on the health systems in many countries, which affecting both patients and health service providers. The rigorous nature of healthcare service management leads patients to try finding a mechanism for faster and better services. It appears that improving the quality of health care services and accurate monitoring of delivery processes, along with some strategies for regulating payroll and medical tariffs, strict rules and regulations and improving health staff motivation would be effective ways for confronting informal payments. Improving the health insurance contribution, promoting transparency & accountability in health system especially in financing, identifying precise control mechanism, using empower patient/public related approach, modifying community perception, reinforcing social resistance to unofficial payments and rebuilt lost social capital in health care are some of the other recommendations in this field.

Since the solutions to control IPs are diverse and complex, ranges of strategies at the country-level are required to rid the health systems of the negative effects of these payments. Consequently, a comprehensive, dynamic, and systemic approach would be a fair tactic on this issue. A key point is that before applying any strategy for IPs, the impact of these payments on access, efficiency, equity, and other goals of the health system should be reconsidered.

## Supplementary information

**Additional file 1.** Appendix-Index of publications included in the review. Index of publications included in this review has been shown in Additional file 1. Each code is a determination for each study which explained by the reference’s number.

**Additional file 2.** The summary of literature review included in this study. The summary of literature review is described in Additional file 2. This summary identified the main characteristics of the included study.

## Data Availability

The datasets used and/or analyzed during the current study are available from the corresponding author on reasonable request.

## Abbreviations

IPs: Informal payments
OOPs: Out-of-pocket payments
UHC: Universal Health Coverage
