# Supplementary material for: New insight into the informal patients’ payments on the evidence of literature: a systematic review study
Source: BMC Health Serv Res. 2020 Jan 6;20:14. doi: 10.1186/s12913-019-4647-3 (PMC6943960; doi:10.1186/s12913-019-4647-3)
Supplement: Supplementary file 1 — Additional file 1. Appendix-Index of publications included in the review. Index of publications included in this review has been shown in Additional file 1. Each code is a determination for each study which explained by the reference’s number. [file 12913_2019_4647_MOESM1_ESM.docx]

| ***References*** | ***Author*** | ***Country*** | ***Study Design*** | ***Sampling*** | ***Result Summary*** |
| --- | --- | --- | --- | --- | --- |
| **Code1** | Aarva P, et al. | Russia | Quantitative | Random sample | The practice of IPs exists alongside the introduction of formal chargeable governmental health services in Russia. Around 15% of respondents (n¼2001) in two Russian cities had made IPs in the past 3 years. Being female, having a chronic disease, being a pensioner, and being willing to pay for additional medical information increased the probability of making IPs for health care services. |
| **Code2** | Balabanova D, et al. | Bulgaria | Qualitative | Random sample | IPs are relatively common in Bulgaria, especially if in the form of gifts. Informal cash payments are universal for operations and childbirth, clear-cut and life-threatening procedures, in hospitals or elite urban facilities or well-known physicians. Most gifts were given at the end of treatment and most cash payments-before or during treatment. Wealthier, better educated, younger respondents tend to pay more often, as a means of obtaining better-quality treatment in a de facto two-tier system. |
| **Code3** | Stepurko T, et al. | Six Central and Eastern European countries | Quantitative | Multi-stage random sample | Health-care users in Bulgaria and Poland are less inclined to make informal payments, while health-care users in Romania and Ukraine most often report such payments. The IP rates for Hungary and Lithuania fall between these two groups. In all six countries, individuals who feel uncomfortable when leaving the physician’s office without a gratuity and who feel unable to refuse the request of medical staff to pay informally, more often make IPs. |
| **Code4** | Mokhtari M, et al. | Moldovan | Quantitative | Random sample | A probability model of IPs shows that knowledge and information have significant impact on reducing frequency of IPs .Thus, informing patients, which is the least expensive and intrusive role of the government, could significantly reduce out-of-pocket health care spending. |
| **Code5** | Bertone MP, et al. | Sierra Leone | Quantitative | Random sample | This study describes the incomes of primary health workers in Sierra Leone and finds that salaries make up about 60% of the total revenues, while the rest is composed by a variety of formal and informal incomes. Health workers’ narratives reveal that the satisfaction related to the incomes does not depends only on their amounts, but also on non-financial features. Based on these features, health workers choose to assign incomes to different uses. These findings have policy implications for designing incentives as they call for more attention to the earning opportunities for health workers beyond formal allowances, and to the HWs own perspectives which question the assumption of income fungibility. |
| **Code6** | Belli P, et al. | Georgia | Qualitative | Purposive sampling | The study finds that the demarcation between formal and informal components of these Out-of-Pocket payments is extremely imprecise because of: (a) the spread of “health rights unawareness” around the country and, (b) the prices outside the BBP differfrom provider to provider. The study also found that some IPs are based on cultural/social patterns ingrained in the Georgian tradition. Georgian now about the low health sector salaries and compensate the doctors with small payments. |
| **Code7** | Najar AV, et al. | Iran | Mix method | Multi-stage sampling | Sixteen (5.93%) patients made voluntary IPs. The purpose of payment was: “gratitude” (43.75%), satisfaction with health services provided” (31.25%) and (18.75%) for better quality of services. About 75% of the payments were occurred during receiving health care services. The main causes were “no request for IPs” (98.14%), “not affording to pay for IPs” (73.33%) and “paying the hospital expenses by taking out a loan” (55.91%). Responders said they would pay informally in demand situation (51.85%) just for patient’s health priority, 40.71% would also “search for other alternative solutions” and 27.33% “accepted the demand as a kind of gratitude culture”. Twenty four patients (8.9%) had experienced mandatory informal payments during the last 6 months. The minimum amount of payment was 62.5$ and the maximum was 3125$. There was a significant relationship between the way of referring to medical centers and informal patient's payment (P ≤0.05). |
| **Code8** | Kaitelidou DC, et al. | Greece | Qualitative | Random sample | Out of the total number of those reporting treatment in public hospitals (N= 336), 36% reported at least one informal payment to a doctor. Of these, 42% reported it was given because of the fear of receiving sub-standard care (if they did not pay) and another 20% claimed that the doctor demanded such a payment. None of the socio-economic characteristics of the family were related to the size of extra (informal) payments. The probability of extra payments is 72% higher for patients aiming to “jump the queue”, compared tothose admitted through normal procedures. Also, surgical cases had a 137% higher probability for extra payments compared to non-surgical patients. |
| **Code9** | Stepurko T, et al. | Bulgaria, Hungary and Ukraine | Quantitative | Multi-stage sampling | A significantly higher number of users report informal payments to a specialist compared to GPs and also significantly more frequent and higher payments are noted for surgery and pregnancy/childbirth except for emergency childbirth.  In- patients pay higher amounts when the reason for the IP is better services as well as when requested by medical staff. In addition to the IPs, in Bulgaria, Hungary and Ukraine, respondents also report that they brought goods for their last hospitalization at the medical staff’s request. Poor knowledge of the formal fee size is associated with higher amounts paid informally for the last physician visit. Poor knowledge of the formal fee size is associated with higher amounts paid informally for the last physician visit. |
| **Code10** | Piroozi B, et al. | Iran | Quantitative | Multi-stage sampling | It seems that the implementation of the health transformation plan (HTP) reduced the mean out-of-pocket (OOP) payments for inpatient services in Kurdistan province, Iran. After the implementation of the third phase of the HTP, during the study period, we did not found any informal cash payments to physicians for inpatient services. Several factors significantly increased the probability of IPs to physician including the followings: being discharged from private sector and social security hospitals, being discharged from hospitals before the implementation of the HTP, living in rural areas, being visited by an ophthalmologist, urologist, or otolaryngologists. |
| **Code11** | Tomini SM, et al. | Albania | Quantitative | Not clear | Findings suggest that vulnerable groups in society remain less protected against such payments and policy measures have not reached the most deprived regions of the country. |
| **Code12** | Gaal P, et al. | Hungary | Review | - | Reviewing the various theories on the causes of IP they have summarized the essence of the debate as revolving around two contrasting hypotheses, ‘donation’ or ‘fee-for-service’, with different consequences for appropriate policy response. Although distilled from the theoretical debates in Hungary, they seem to be relevant to other countries facing IP, and consequently th  ey have a wider applicability. |
| **Code13** | Jahangiri R, et al. | - | Systematic review | - | Thirty-three full papers were selected for study and analysis. Studies described different characteristics for IPs, so there was no uniform definition for this phenomenon. The causes of informal payments were categorized into three groups: payers wish to receive higher quality services, better access or in order to saving time; Second, from the caregivers’ perspective was low wages and tariffs, and irregular deductions from insurance companies; Finally, the health system's weaknesses include insufficiency of insurance coverage, inadequate monitoring of tariffs and the lack control over charges and lack of human resources in some cases. |
| **Code14** | Aboutorabi A, et al. | Iran | Quantitative | Random sample | The results indicated that 21% (n=63) of individuals paid informally to the staff. About 4% (n=12) of the participants were faced with informal payment requests from hospital staff. There was  a significant relationship between frequency of informal payments with marital status of participants and type of hospitals. According to our findings, none of the respondents had IPs to physicians. The most frequent informal payments were in cash and were made to the hospitals’housekeeping staff to ensure more and better services. There was no significant relationship between the informal payments with socio-demographic characteristics, residential area and insurance status. |
| **Code15** | Baji P, et al. | Hungary | Quantitative | Multi-stage  random | They identified three main different attitudes towards IPs: accepting informal payments, doubting about informal payments and opposing IPs. Those who accept informal payments (mostly young or elderly people, living in the capital) consider these payments as an expression of gratitude and perceive them as inevitable due to the low funding of the health care system. Those who doubt about informal payments (mostly respondents outside the capital, with higher education and higher household income) are not certain whether these payments are inevitable, perceive them as similar to corruption rather than gratitude, and would rather use private services to avoid these payments. They found that the opposition to IPs (mostly among men from small households and low income households) can be explained by their lower ability and willingness to pay. |
| **Code16** | Tambor M, et al. | Mix in European countries | Review | - | The results suggest that the presence of obligatory cost-sharing for health care services is associated with governance factors, while informal patient payments are a multi-cause phenomenon. |
| **Code17** | Kpanake L, et al. | Togo | Quantitative | Convenience sample | A substantial minority of participants rejected the practice of informal payments, irrespective of circumstances. In contrast, a majority of them were of the opinion that when patients were wealthy and physicians were underpaid, this practice was acceptable. Health practitioners more frequently adhered to the ethical position than lay people. Implications for health policy are discussed. |
| **Code18** | Skordis-orrall J, et al. | India | Quantitative | Not clear | High expenditure as a proportion of household resources should alert policymakers to the burden of maternal spending in this context. Differences in IPs, significantly regressive indirect spending and the use of savings versus wages to finance spending, all highlight the heavier burden borne by the most poor. If a policy objective is to increase institutional deliveries without forcing households deeper into poverty, these inequities will need to be addressed. |
| **Code19** | Nekoeimoghadam M, et al. | Iran | Qualitative | Purposive sampling | They found that people make IPs for several reasons, namely cultural, quality-related and legal. Providers ask for IPs because of tariffs, structural and moral reasons, and to demonstrate their competence. Informal payments were found to be more prevalent for complex procedures and are usually asked for directly. |
| **Code20** | Vian T, et al. | Albania | Qualitative | Purposive sampling | The results suggest that factors promoting IPs in Albania include perceived low salaries of health staff; a belief that good health is worth any price; the desire to get better service; the fear of being denied treatment; and the tradition of giving a gift to express gratitude. Members of the general public also believe IPs create uncertainties and anxiety during the care-seeking process, while providers perceive that informal payments harm their professional reputation, induce unnecessary medical interventions, and create discontinuity of care. |
| **Code21** | Thompson R, et al. | - | Review | - | It is clear that an entrenched IP system is difficult to address and will remain a necessary component of health sector spending in the short term. On the one hand IPs appear to be a rational response to the deficiencies of health care systems in the transitional economies, yet they also represent a failure to capture valuable revenue which could be targeted towards health policy objectives. The limited evidence available suggests that they create perverse financial incentives and that, despite reports of price discrimination, they also have a negative impact on equity. |
| **Code22** | Vian T, et al. | Moldova | Mix method | Convenience sampling | Efforts to expand financial protection should focus on reducing household spending on medicines and hospital-based IPs. Reforms should consider ways to reduce medicine prices and promote rational use, strengthen administrative controls, and increase incentives for quality health care provision. |
| **Code23** | Vian T, et al. | Albania | Qualitative | Convenience sampling | Comparing people who intend to make IPs with people who do not intend to make payments, the study found differences in attitudes  as well as beliefs about the consequences of making IPs, in perceptions about what others think and in control beliefs, but no difference in moral beliefs or demographic characteristics.  People who intend to make IPs the next time they seek care are more likely to believe they will get faster and better quality care than non-intenders, but also think they must pay to receive any care at all. People who do not intend to make IPs are more likely to report that  they have connections with medical personnel, which may be substituting for IPs. |
| **Code24** | Tomi S, et al. | Albania | Quantitative | Random sample | Results show that medical staff has less information on the patients’ maximum willingness to pay informally than patients have on medical staff’s minimum expected amount. These estimates do not depend on categories of illnesses but on certain sociodemographic characteristics |
| **Code25** | Tengilimoğlu D, et al. | Turkey | Quantitative | Random sample | The data analysis revealed that approximately 29% of the study participants made IPs in return for the medical service they received. Three out of 4 people who made IPs were from a low-income group. IPs were made in the form of cash prior to medical procedures and also as gifts following the procedures. |
| **Code26** | Stepurko T, et al. | Ukraine | Qualitative | Convenience sampling | The results suggest that IPs for childbirth are an established practice in Kiev maternity hospitals. The bargaining process between the pregnant woman (incl. her partner) and the obstetrician is an important part of the predelivery arrangement, including the IP. |
| **Code27** | Mejsner SB, et al. | Western Balkans countries | Review | - | Research has reported incidents of IPs on a wide scale and has described various patterns and characteristics of these payments. Although these payments have typically been small – particularly to providers in common areas of specialized medicine – evidence regarding bought and brought goods remains limited, indicating that such practices are likely even more common, of greater magnitude and perhaps more problematic than IPs. |
| **Code28** | Baji P, et al. | Hungary | Quantitative | Random sample | 9% of the patients paid informally during their last visit to GP (2 Euros on average), 14% paid informally for specialist care (35 Euros on average) and 50% paid informally for hospitalisation (58 Euros  on average).They found a significant reduction in the probability of paying informally only for elderly patients in case of in-patient care. Results suggest that IPs are widely spread in Hungary, especially in in-patient care. |
| **Code29** | Atanasova E, et al. | Bulgaria | Quantitative | Multi-stage random | IPs continue to exist in Bulgaria even 10 years after the implementation of official co-payments for services included in the basic benefits package. Although most respondents are against the practice of IPs in cash, gifts in kind are still seen positively by a sizeable minority. Although most respondents are against the practice of IPs in cash, gifts in kind are still seen positively by a sizeable minority. |
| **Code30** | Burak LJ, et al. | Albania | Quantitative | Convenience sample | The constructs of the theory explained 34% of the variance in intentions to make under-the-table payments, with attitude toward the behavior making the strongest contribution. Using the TPB to examine the practice of making under-the-table payments has indicated specific areas that can be targeted by policy interventions. |
| **Code31** | Liu T, et al. | - | Review | - | IPs for health care in the countries of Central and Eastern Europe and China are widespread. They are widely condemned on moral grounds and governments in those countries are urged by the public to take effective actions to ban IPs. |
| **Code32** | Liaropoulos L, et al. | Greece | Quantitative | Random sample | Out of the total number of those reporting treatment in public hospitals (N= 336), 36% reported at least one IP to a doctor. Of these, 42% reported it was given because of the fear of receiving sub-standard care (if they did not pay) and another 20% claimed that the doctor demanded such a payment. None of the socio-economic characteristics of the family were related to the size of extra (informal) payments. The probability of extra payments is 72% higher for patients aiming to “jump the queue”, compared to those admitted through normal procedures. Also, surgical cases had a 137% higher probability for extra payments compared to non-surgical patients. |
| **Code33** | Riklikiene O, et al. | Lithuania | Quantitative | Random sample | Users of healthcare services usually made IPs for the visit to, and consultation with, a general practitioner or physician specialist and for diagnostic services. Females and older citizens, the disabled, public/private sector employees, retirees, those with higher education and those in the highest household monthly income group were more prone to pay informally for health services. The majority of respondents were against formalization of IPs. |
| **Code34** | Stepurko T, et al. | Lithuania, Poland and Ukraine | Quantitative | Stratified random sample | The empirical results suggest a lower share of IPs as well as a prevalence of more negative attitudes towards informal patient payments in Poland compared to Lithuania and Ukraine. Informal payments are more common and more expensive for in-patient health care services in contrast to out-patient ones in all countries.  Still, in post-Soviet Lithuania and Ukraine informal patient payments co-exist with other types of patient payments such as quasi-formal patient payments. |
| **Code35** | Khodamoradi A, et al. | Iran | Quantitative | Random sample | Given the results of this study that indicated high amounts of IPs. Type of hospital, place of settlement, treatment procedure & income were recognized as the related factors with the amount of IPs. |
| **Code36** | Stepurko T, et al. | Six Central and Eastern European countries | Quantitative | Stratified random sample | Overall, around 35–60% of the general public in each country has ever made informal payments, though informal cash payments are perceived negatively, mostly as corruption. In-kind gifts are often seen as a token of gratitude. However, significant differences among countries are observed. Despite the public support for the eradication of IPs, there are population groups who favor their existence and this should be taken into account in policy-making. |
| **Code37** | Maduke T, et al. | In 95 low & middle income countries | Report | - | Corruption is a debilitating force that weakens the quality and vitality of health services. It is a complex and pervasive challenge in the health sectors of Low and Middle Income Countries . However, many things can be done to reduce corruption and protect public health sector from the scourge of corruption. The causes and strategies outlined in this document are only a start; more examination is needed to better understand the root causes of corruption and the means to address them. |
| **Code38** | Lewis M, et al. | - | Report | - | This working paper looks at factual evidence to describe the main challenges facing health care delivery in developing countries, including absenteeism, corruption, informal payments, and mismanagement |
| **Code39** | Tomini S, et al. | Albania | Quantitative | Stratified sample | Findings suggest differences in determinants of informal payments in inpatient and outpatient care.  Generally our results show that IPs are dependent on certain characteristics of patients, including age, area of residence, education, health status and health insurance. However, they are less dependent on income, suggesting homogeneity of payments across income categories. |
| **Code40** | Cohen N, et al. | Israel | Quantitative | Proportional, stratified sampling | They found that IPs do exist in Israel—although it seems that there has been a decline in the phenomenon.Contrary to the literature, they find no relationship between the option of voice or dissatisfaction with healthcare services and IPs. However, we do find a negative correlation between trust and the use of such payments. This finding is consistent with Hirschman’s insight that a lack of loyalty may lead people to strategies of exit. They suggest that given the fact that health care in Israel is a public service, the exit option may actually be a quasi-exit behavior |
| **Code41** | UNGUREANU MI, et al. | Romania | Quantitative | Not clear | Out of the total sample population, 44.2% believe that in the inpatient surgery care services gifts are offered most frequently, while 55.31% of respondents place inpatient surgery care as being the place where highest amounts of money are given. This  may represent a threat for people’s access to this type of services, as well as for the equity of care across different socio-economic categories. |
| **Code42** | Zhu W, et al. | China | Quantitative | Not clear | The authors argued that medical professionals’ choice of taking “red envelopes” is actually more a way to compensate for their problematic self-image and marred dignity in real practice. Medical professionals in China as a whole are in an embarrassing situation where the work pressure and income, and the sense of pride that used to be part of their profession are not comparable to each other. Under this circumstance, we believe that the effective way to deal with the “red envelopes” issue does not lie solely in introducing more stringent regulations or granting medical professionals  higher payments, but rather in protecting and enhancing the professional dignity of all those working in healthcare. |
| **Code43** | Mohammad S, et al. | Iran | Quantitative | Convenience sample | All the persons studied had an experience of OOP. Formal payments included the free cost of the services with no insurance cover and the margin between the tariff and the rate that the physician or the hospital declares (83% of OOP). In addition 10% of persons encountered informal payments 80% of which was due to the physician’s request and 20%as a gift. The results describes the OOP`s side effects so that 52% of patients faced a postpone in getting services because of the OOP and 72% pointed that OOP has had an enormous effect on providing the necessary needs of the families. |
| **Code44** | Kankeu HT, et al. | Cameroon | Quantitative | Random sample | Results reveal that circa 3.05% of the surveyed patients incurred informal payments for the consultations made on the day of the survey. The amount paid informally represents up to four times the official tariff. Factors related to the following: (i) human resource management of the health facilities (e.g., task shifting); (ii) health professionals’ perceptions vis-à-vis the remunerations of HIV care provision; and (iii) reception of patients (e.g., waiting time) significantly influence the probability of incurring IPs. Also of note, the type of healthcare facilities is found to play a role: IPs appear to be significantly lower in private non-profit facilities compared with those belonging to public sector. |
| **Code45** | Williams CC, et al. | East-Central Europe | Quantitative | Multi-stage random sample | Finding is that patients in Hungary, Latvia, Lithuania, Slovakia, Bulgaria and Romania are significantly more likely to make extra IPs or to give valuable gifts to medical practitioners or to make a hospital donation additional to the official fees. Women are more likely to make IPs for healthcare services whilst unemployed patients or those never or almost never having difficulties in paying bills are less likely to make IPs. |
| **Code46** | Uka, A. | Kosovo | Qualitative | Purposive sampling | The results show that informal cash payments are common for surgeries and childbirth and skipping waiting lines for diagnostic tests. Paying informally seems more likely to be a result of culture and tradition rather than socio-economic conditions. |
| **Code47** | Hasanpoor E, et al. | Iran | Report | - | IPs have adverse impact on access and utilization of health services, efficiency, quality and equity. IPs lead to false information about the real costs of disease and the patient share of these costs and consequently wrong government policies. |
| **Code48** | Kankeu HT, et al. | 33 African countries | Quantitative | Random sample | We obtained that: i) the socioeconomic gradient in IPs is in favor of the rich in almost all countries, indicating a rather regressive system; ii) this is mainly due to the socioeconomic disadvantage itself, to poor/rich differences in supply side factors like lack of medicines, absence of doctors and long waiting times, as well as regional disparities |
| **Code49** | Meskarpour-Amiri M, et al. | Iran | Quantitative | Random sample | About 48% of respondents reported at least one experience of IP for health care during the previous year. The results showed that the patients’ socioeconomic status can significantly affect the likelihood and frequency of IPs for health care. Older people, members of small and wealthier families, employed persons, and those who are under coverage of only basic medical insurance are more at risk of making such payments. |
| **Code50** | Habibov N, et al. | 29 transitional countries | Quantitative | Random sample | They found that being from a wealthier household, experiencing lower quality of healthcare in the form of long waiting times, lack of medicines, absence of personnel, and disrespectful treatment, and having relatives to help when needed, are associated with a higher odds ratio of IPs. Conversely, working for the government is associated with a lower odds ratio of IPs. Living in the countries of the former Soviet Union and in Mongolia is associated with the highest likelihood of IPs, and this is followed by the countries of the Southern Europe. In contrast, living in the countries of Eastern Europe is associated with the lowest likelihood of IPs. |
| **Code51** | Özgen H, et al. | Turkey | Quantitative | Random sample | Using multivariable logistic model, household health expenditure, health insurance, service type and provider ownership were found to be statistically significant predictors of IP. The findings suggest the supply side factors as the main drivers of IPs and thus a need for radical changes in the supply side as well as actions to rebuild lost confidence in the health care system of Turkey. |
| **Code52** | Belli P, et al. | Georgia | Qualitative | Not clear | The extent of direct payments for health services are producing severe consequences on both equity and efficiency, making services unaffordable for most people, and leading to under funding of essential inputs. |
| **Code53** | Cao, X. | China | Quantitative | Not clear | Data collected from two hospitals show that doctors’ misbehaviour is closely related to a number of structural issues embedded in contemporary China’s public health services – funding issues, payment systems, corruption and motivation – but more importantly the study demonstrates the rationality of employee fiddles and management responses. Findings indicate that doctors are mainly responsible for this fiddling, unethical and illegal activity because of the financial gains acquired from patient bribery. |
| **Code54** | Tatar M, et al. | Turkey | Quantitative | Two-stage stratified cluster sample | We concluded that informal payments in Turkey are significant and have important implications for health care reform. |
| **Code55** | Souliotis K, et al. | Greek | Quantitative | Multi-stage random sample | The survey reports under-the-table payments for approximately 32.4 % of public hospital admissions. Private clinics, which display the bulk of out-of-pocket payments, naturally display the lowest under-the-table payments. The highest percentage of under-the-table payments in the private sector appears at visits to private practitioners and dentists (36 %). IPs are most frequently made upon request, prior to service provision, to facilitate access to care and to reduce waiting times, and at a much lower percentage, to post-service provision, and out of gratitude. |
| **Code56** | Gordeev VS, et al. | Russia | Quantitative | Multi-stage random sample | They present the scale and scope of IPs, as well as patterns of IPs and their determinants. They discuss the reasons for discrepancies in estimations and implications for the ongoing reforms. |
| **Code57** | Ensor T. | - | Review | - | This paper considers the prevalence of unofficial payments in transitional economies, the impact they have on the health sector and individual access to health and the possible policy strategies that could be adopted to address them. |
| **Code58** | Ensor T, et al. | Kazakstan. | Review | - | Estimates on contributions to the costs of medicines in Kazakstan suggest that they may add 30% to national health care expenditure. Payments to staff are likely to add substantially to this figure, although few reliable statistics exist. Research in this area is important since IP is likely to impact on equity in access to medical care and the efficiency of provision. The impact of attempts to reform systems using Western ideas could be reduced unless account is taken of the effect and size of the informal payment system. |
| **Code59** | Parsa M, et al. | Iran | Qualitative | Purposive sampling | Six topics were extracted from the interviews including definitions, commonness, varieties, motivations, outcomes and preventive measures. It was revealed that under-the-table payments are the money taken (either in pri-vate or public portions) from patients in addition to what formally is determined. This problem is mostly seen in sur-gical services and the most important reason for it is unrealistic tariffs. |
| **Code60** | Agheorghiesei D-T, et al. | Romania | Qualitative | Deductive approach | The gift practice in Romanians is a national-cultural feature intensely debated by several authors. Their assumption is that this cultural specificity in conjunction with other factors (for instance, specific values, such as safety, sacrifice, hospitality, kindness, tolerance, but also the hierarchical submission or the importance of the group opinion, the attitude towards the risk) can be factors which explain the practice and the magnitude of the IPs phenomenon in the autochthonous medical system. |
| **Code61** | Lewis M. | - | Review | - | With minimal funding levels and limited accountability, publicly financed and delivered care falls prey to illegal payments, which require payments that can exceed 100 percent of a country’s median income. Methods to address the abuse include establishing official fees, combined with improved oversight and accountability for public health care providers, and a role for communities in holding providers accountable. |
| **Code62** | Pavlova M, et al. | - | Review | - | This paper outlines the negative effects of IPs for public health care provision and the reasons for their existence. Based on this, the paper discusses a mixture of strategies as a plausible solution to informal patient payments. The focus is on policy mechanisms that can help to deal with this type of payment in a country. |
