# Supplementary material for: New insight into the informal patients’ payments on the evidence of literature: a systematic review study
Source: BMC Health Serv Res. 2020 Jan 6;20:14. doi: 10.1186/s12913-019-4647-3 (PMC6943960; doi:10.1186/s12913-019-4647-3)
Supplement: Supplementary file 2 — Additional file 2. The summary of literature review included in this study. The summary of literature review is described in Additional file 2. This summary identified the main characteristics of the included study. [file 12913_2019_4647_MOESM2_ESM.docx]

| ***Code (Reference)*** | | | | | | |
| --- | --- | --- | --- | --- | --- | --- |
| Code1 (51) | Code11 (93) | Code21 (27) | Code31 (8) | Code41 (38) | Code51(85) | Code61 (7) |
| Code2 (39) | Code12 (35) | Code22 (66) | Code32 (10) | Code42 (94) | Code52 (63) | Code62 (74) |
| Code3 (65) | Code13 (45) | Code23 (60) | Code33 (52) | Code43 (88) | Code53 (72) |  |
| Code4 (37) | Code14 (49) | Code24 (26) | Code34 (79) | Code44 (40) | Code54 (48) |  |
| Code5 (24) | Code15 (32) | Code25 (54) | Code35 (61) | Code45 (55) | Code 55 (43){Souliotis, 2016 #205} |  |
| Code6 (23) | Code16 (25) | Code26 (53) | Code36 (13) | Code46 (77) | Code56 (95) |  |
| Code7 (17) | Code17 (36) | Code27 (71) | Code37 (42) | Code47 (47) | Code57 (73) |  |
| Code8 (96) | Code18 (82) | Code28 (84) | Code38 (97) | Code48 (41) | Code58 (90) |  |
| Code9 (98) | Code19 (18) | Code29 (31) | Code39 (59) | Code49 (16) | Code59 (83) |  |
| Code10 (50) | Code20 (62) | Code30 (33) | Code40 (34) | Code50 (14) | Code60 (70) |  |
